# Editorial: Engineered cell-originated biomimetic materials for cancer therapy

**DOI:** 10.3389/fbioe.2023.1259959

**Published:** 2023-07-27

**Authors:** Lan Xiao, Yang Zhang, Mengya Zhang, Jie Gao

**Affiliations:** ^1^ School of Medicine and Dentistry, Griffith University, Gold Coast, QLD, Australia; ^2^ Centre for Biomedical Technologies, Queensland University of Technology, Brisbane, QLD, Australia; ^3^ Nanomedicine and Intestinal Microecology Research Center, Shanghai Tenth People’s Hospital, School of Medicine, Tongji University, Shanghai, China; ^4^ Changhai Clinical Research Unit, Shanghai Changhai Hospital, Naval Medical University, Shanghai, China

**Keywords:** cancer immunotherapy, cancer vaccine, nanovaccine, cell membrane camouflage, biomimetic material, engineered nanoparticles

Cancer remains one of the leading causes of death worldwide and a major obstacle to increasing human life expectancy. The traditional cancer therapies such as chemotherapy and radiotherapy (in combination with surgical removal) are not satisfactory owing to the limitations such as damage on the healthy tissue/organ, systemic toxicity, and drug-resistance, calling the urgent need for advanced therapeutical approaches that can specifically target the tumor cells without affecting the normal ones (Zhong et al., 2023). Recently, the engineered cell-originated biomimetic materials have emerged as attractive cancer therapeutic agents (Zeng et al., 2022; Zhang et al., 2023). Engineering modifications can enhance the biological properties such as tumor-targeting and immunogenicity, thus increasing drug accumulation in tumor or enhancing the effect of immunotherapy against tumor. In addition, drawbacks of the synthetic materials (e.g., low biocompatibility, accumulation in immune cells other than the tumor cells) can be resolved through this combination of cellular components and nanomaterials (Zeng et al., 2022; Zhang et al., 2023). Therefore, the engineering of cell-originated biomimetic materials is an advanced strategy to develop innovative, biocompatible, and multifunctional biomaterials with translational potentials in tumor therapy.

The aim of this Research Topic was to broaden the knowledge and application of the engineered cell-originated biomimetic materials for tumor therapeutics, especially the novel engineering strategies to improve the immunotherapeutic efficacy of cancer vaccines, sensitize conventional oncology therapies, enhance drug delivery efficiency, or provide insight into the therapeutic mechanisms of materials. The issue currently includes 4 papers, including two research papers on the development of cellular membrane-engulfed nano-drug delivery system for cancer raidochemotherapy, the regeneration of T cells for CAR-T cancer treatment, and two review papers to summarize the recent advances on biomembrane-engulfed nanomaterials for anti-tumor gene delivery, and on the design of anti-cancer scaffolds. The papers cover multidisciplinary research from several fields such as oncology, immunology, material science, nanotechnology, and biomedical engineering.

The paper Du et al. developed an advanced nanosystem for potential radiochemotherapy against osteosarcoma. In this work, to enhance the radiotherapy effect and minimize the side-effect of chemotherapy drug, a pH-sensitive nanomaterial [metal-organic framework zeolite imidazole framework-8 (ZIF-8)] was used to carry Dbait (radiosensitizer) and Adriamycin (the chemotherapy drug), therefore ensure a tumor-environment-specific drug release. Moreover, a hybrid platelet-osteosarcoma cell membrane (OPM) was used to coat the ZIF-8 to obtain the nanosystem Dbait-ADM@ZIF-8]OPM, therefore facilitating the targeting on tumor cells (via osteosarcoma cell membrane) and preventing the phagocytosis of the nanosystem by phagocytes (via platelet membrane). Such an advanced nanosystem showed efficient anti-tumor effects *in vivo* with no obvious biotoxicity, suggesting its translational potential in osteosarcoma treatment.

The paper Chen et al.” provided a flexible approach to develop functional clusters of the differentiated (CD8^+^) T cells from human induced pluripotent stem cells (hiPSC) for chimeric antigen receptor (CAR) T cell treatment, therefore significantly benefiting the CAR-T immunotherapy against cancer. The authors developed an Ff differentiation technique for generating the hiPSCs-based T cells, then facilitated their maturation into CD8^+^ hiPS-T cells. After that, the cells were infected with CD19 CAR lentivirus to obtain the hiPS-CAR-T cells, and the obtained CAR-T cells showed high antigen-specific cytotoxicity (similar to the CAR-T harvested from peripheral blood) and significant anti-tumor capacity *in vitro* and *in vivo*. This work therefore provides a translational technique to generate CAR-T cells from the iPSC for cancer treatment.

The two review papers summarize the latest advances on anti-cancer nanomaterials and scaffold materials. The review Li et al. focused on the progress in nanomaterial-based gene delivery system for cancer treatment. Gene delivery has been considered a promising approach in tumor control. However, there are certain limitations such as the high-degradability of the naked nucleic acid molecules, the cytotoxicity of nuclear transfection and electroporation, and other issues with biodistribution and tumor-specific targeting, which significantly hinder the application of gene therapy against cancer, suggesting the need for a carrier capable of protecting the nucleic acid molecules and targeting the tumor cells with optimal biocompatibility. The development of biomembrane-wrapped nanoparticles (MBNPs) can serve as ideal candidates for these purposes because of their low immunogenicity, long circulation time, and tumor specific targeting owing to the biomimetic coating, with the nanocarrier to protect the gene cargos, therefore significantly improving the efficacy of gene delivery and precenting side-effects. This review summarized the latest advances on the technique for MBNP development and the future challenges, hence providing critical guides for the design of gene delivery platform in tumor therapy and prospects of gene delivery MBNPs toward clinical transformation are introduced. The principal purpose of this review is to discuss the biomedical potential of gene delivery MBNPs for cancer therapy and to provide guidance for further enhancing the efficiency of tumor gene therapy. The review Schluck et al. summarized the design parameters for scaffolds to enhance their anti-tumor performance. The biomaterial scaffolds are considered as promising approaches for cancer vaccination and adoptive cell transfer (ACT) for cancer treatment, as they provide a 3D environment consisting of anti-tumor immune niches to deliver cancer vaccines or ACTs at specific implantation sites. The host responses are the major challenges in scaffold-based cancer immunotherapy because the properties of scaffolds such as surface chemistry, biodegradability, mechanical property, porosity and interconnectivity of the pores can affect the local immune environment to and impair the therapy effect. This review provides an overview of the effects of these properties on cancer vaccine and ACT delivery and highlighting the ideal properties for the design of anti-cancer scaffolds.

Overall, this Research Topic recruited novel technologies and critical insights in the anti-cancer strategies which are expected to benefit cancer research and treatment in the future.
